# Evaluation of the Anxiolytic Potential and Tolerability of a CBD-Rich Cannabis Extract in Adults with Anxiety: Results from a Single-Arm Clinical Trial

**DOI:** 10.1192/j.eurpsy.2026.11232

**Published:** 2026-07-31

**Authors:** I. T. Moreira, M. D. S. Sousa, J. S. Barbosa, C. C. M. R. De Brito, V. R. D. Neves, M. E. A. Fernandes, A. D. S. Coêlho, R. M. H. Formiga, J. M. D. A. Batista, L. A. M. Monteiro, E. A. D. S. Júnior

**Affiliations:** Federal University of Paraíba, João Pessoa, Brazil

## Abstract

**Introduction:**

Anxiety disorders affect a substantial portion of the global population and represent one of the leading causes of disability worldwide, being strongly associated with reduced quality of life and significant psychosocial and economic burden. Cannabidiol (CBD), a non-psychoactive phytocannabinoid derived from Cannabis, has emerged as a promising compound with potential anxiolytic properties. Preclinical and clinical studies suggest that CBD may modulate anxiety-related responses through interaction with the endocannabinoid and serotonergic systems, supporting its therapeutic potential in anxiety management.

**Objectives:**

This study aimed to evaluate the efficacy and safety of short-term oral administration of a CBD-rich cannabis extract in adults with anxiety symptoms, to explore general patterns of dose tolerance and to monitor common treatment-related adverse effects.

**Methods:**

A prospective, single-arm clinical trial was conducted at the Federal University of Paraíba, Brazil, with approval from the Research Ethics Committee of the Center for Medical Sciences. The participants underwent an individual medical consultation before the intervention and after 8 weeks of treatment, in which the Beck Anxiety Inventory (BAI) was administered to assess anxiety symptoms index. Participants received escalating oral doses of a CBD-rich cannabis extract, starting at 50 mg/day and increasing by 50 mg every two weeks, up to a maximum of 200 mg/day, when well tolerated. Otherwise, the highest well-tolerated dose was maintained until the end of the intervention, and outcomes were assessed regardless of this adjustment. Statistical analyses used paired *t*-tests or Wilcoxon signed-rank tests, with significance set at *p* < 0.05

**Results:**

Among 65 enrolled participants (mean age = 35 ± 11.8 years; 72.3% female), 14 discontinued the treatment (6 due to intolerance, 8 due loss follow-up). Mean BAI scores decreased from 35.7 ± 8.8 to 12.2 ± 10.6, with severe anxiety falling from 74.5% to 21.6%. Intention-to-treat analysis confirmed significance (mean difference: 24.0; CI 95%: 20.4 - 27.8; *p* < 0.001).Participants using concomitant psychotropic drugs showed greater improvement (mean difference = 27 vs. 21). The most tolerated dose that complete the protocol was 200mg (n=26) followed by 150mg (n=10), 50mg (n=8) and 100mg (n=7). Common adverse effects included nausea (7), somnolence (6), diarrhea (4),mild gastrointestinal discomfort (3) and dizzines (2).

**Conclusions:**

Short-term administration of cannabidiol markedly reduced anxiety symptoms, even among participants who did not reach the maximum 200 mg/day dose, highlighting its therapeutic potential. Despite some loss to follow-up and dose-limiting tolerability in a few cases, the overall improvement supports CBD as a promising intervention for anxiety, warranting larger controlled trials to confirm and refine these findings.

**Disclosure of Interest:**

None Declared

